# Wind speed interannual variability with measured data validations and its impact on energy yield in the Southwest Sea of Korea

**DOI:** 10.1038/s41598-025-18914-x

**Published:** 2025-09-29

**Authors:** Geonhwa Ryu, Okan Sargin, Dohee Lee, Hyojeong Kim, Anna Pulo, Hyun-Goo Kim, Chang Ki Kim, Chae-Joo Moon, Jin-Young Kim

**Affiliations:** 1 OWC, Wind & Site, Seoul, 04520 Republic of Korea; 2 Orangewind GmbH, 12053 Berlin, Germany; 3 OWC, Wind & Site, 60313 Hamburg, Germany; 4 Korea Institute of Energy Research (KIER), Renewable Energy Institute, Daejeon, 34129 Republic of Korea; 5 Korea Institute of Energy Research (KIER), Renewable Energy Big Data Laboratory, Daejeon, 34129 Republic of Korea; 6 Department of Electrical Engineering, Mokpo National University, Mokpo, 58554 Republic of Korea

**Keywords:** Wind energy, Renewable energy

## Abstract

The Republic of Korea (ROK) has set an ambitious goal of 40.7 GW of onshore and offshore wind farms by 2038, as outlined in the “11th Basic Plan for Electricity Supply and Demand” in May 2024. To achieve this target, both wind farm developers and the government are crafting policies for “Project Site Planning” and “Offshore Wind Farm Clusters”. A major concern for stakeholders is the variability in annual wind resources driven by climate phenomena and climate change, which can lead to increased uncertainty in energy yield predictions. This study investigated the interannual variability (IAV) of wind speed in the Southwest Sea of Korea, a critical factor influencing energy yield predictions and the levelized cost of energy (LCOE) for offshore wind farms. To achieve this goal, we used reanalysis datasets validated against measurement data to calculate a ROK-specific IAV. Our findings indicate the possibility of reducing LCOE estimation by accurately accounting for regional IAV, particularly in Jeonnam, where 14 GW of offshore wind capacity is projected. This research provides essential data for improving energy yield predictions, thereby enhancing financial confidence and supporting the development of expertise in the growing wind energy sector in the ROK.

## Introduction

As climate change caused by indiscriminate greenhouse gas emissions has become more severe, natural disasters such as droughts, storm surges, floods, and heat waves have become more frequent, and countries have had to actively participate in measures to reduce greenhouse gases. Accordingly, in countries and cities where energy consumption is concentrated, large amounts of budgetary investment and policy collaboration are being made for energy conservation and carbon emission reduction measures, such as Carbon-Zero and RE100.

In December 2017, the Republic of Korea (ROK) set a goal to build 12 GW of offshore wind power facilities by 2030 under the “Renewable Energy 3020 Policy” and raised the target to 16.8 GW in December 2020 in accordance with the “5th Basic Plan for the Development and Use of New and Renewable Energy Technology”^1–3^. Subsequently, the target was adjusted to 14.3 GW by the “10th Basic Plan for Electricity Supply and Demand” established in January 2023, and the Ministry of Trade, Industry, and Energy recently announced that this plan will promote the deployment of renewable energy power generation facilities with an average annual capacity of 6 GW by 2030. However, as of the first half of 2024, the installed capacity of offshore wind power facilities currently in operation is less than 1% of the target of the 10th Basic Plan for Electricity Supply and Demand. GWEC (Global Wind Energy Council) estimates that the growth potential of the ROK offshore wind market will increase by 35% over the next five years, following a revision in 2023^4^ that updated the market capacity target to 2.3 GW compared to the projection in 2022^5^. This updated target is based on the acquisition of EBLs (Energy Business License) by 90 domestic offshore wind projects, which represent approximately 27.7 GW of total rated capacity; most of these projects are still in the preliminary stages^6^. This finding suggests the potential for significant growth in the domestic offshore wind power market in the coming years. Expectedly, improvements in industrial infrastructure, a reduction in LCOE (Levelized Cost of Energy, which represents the average total cost of building and operating a power generation facility per unit of electricity generated), and an increase in the skilled workforce and expertise within the sector are expected^7–10^.

The uncertainty of offshore wind projects is evident from the initial stage of a project, which is partly due to the use of wind resource data to determine project feasibility and annual energy production (AEP) estimations. By following industry best practices and guidelines, the use of reliable wind resource data and AEP analysis methods can significantly mitigate this uncertainty. Moreover, several studies have recently shown that climate change can lead to localized variability in wind resources in the long term^11–14^. This is due to mid-long-term changes in atmospheric stability, abnormal increases in sea surface temperature compared to those in the average year, changes in radiation and emission (flux), and changes in jet flow patterns, resulting in a trend of decreasing or increasing wind speeds in different regions^2,13–19^. This will cause an increase or decrease in the amount of wind power generation in the region, which in turn will determine the general and economic feasibility of wind power projects.

The wind speed (WS) and resulting power generation of wind turbine generators (WTGs) vary between regions depending on the temporal and spatial scale. Short-term or mid-long-term prediction of wind speeds near WTG hub heights can have a key impact on site selection, grid management and business economics evaluation. Based on improved prediction accuracy achieved through numerical prediction models and statistical postprocessing, monthly and seasonal information has been increasingly used in wind farm design and planning. However, short-term or mid-long-term predictions of wind speeds are significantly less predictable in regions with more complex terrains and more changes and interactions in meteorological factors^20^. In addition, due to intensifying climate change in recent years, the annual volatility of wind resources has become more prominent than it was in the average year. The interannual variability (IAV) in wind speed refers to the change in wind speed from one year to the next within a specific region. It represents the year-to-year fluctuations in wind speed, as indicated in Eq. 1 (Eq. 1). In particular, the IAV is an indicator of variability in terms of the standard deviation of the average wind speed per year from the long-term average value, which helps to understand the pattern of fluctuations in wind speed by analyzing how the average wind speed varies annually.

1 $$\:IAV=\:\:\frac{{\sigma\:}_{annual\:wind\:speed}}{{V}_{long-term\:mean}}\left[\%\right]$$

In particular, IAVs can have a significant impact on onshore and offshore wind power generation, as wind speed variability affects farm power output and operational efficiency. This is because the expected variability in wind power plays a key role in determining project financing. The impact of IAV on wind power generation is as follows:

- Fluctuations in annual energy production: IAV leads to variations in annual energy generation, introducing challenges for accurate yearly AEP assessment required for project financing and for servicing debt throughout the financing period after construction.
- Uncertainty in power generation forecasting and usage planning: In the regional context, the variability in wind resources introduces uncertainty in forecasting capacity and planning for power usage within the grid.
- Increased operating and maintenance costs: The unpredictable nature of wind speeds due to IAV may lead to higher operating and maintenance expenses, as equipment may need more frequent adjustments and repairs to accommodate varying conditions.

Ultimately, IAV can introduce business risks for wind projects, especially when the full extent of the risk associated with IAV is not fully understood through short-term wind resource measurements from meteorological masts or lidars. This approach is equivalent to asking what errors might arise if a year of measurement data is obtained from a potential wind farm site and assuming that these data represent the farm’s wind resources for the next 20–30 years of operation. To address this issue and enhance the understanding of the associated uncertainty, a detailed local analysis of wind resources is necessary. The results of this analysis should be incorporated into uncertainty assessments to improve the accuracy of annual power generation predictions.

In this regard, Garrad Hassan, the predecessor of the DNV (Det Norske Veritas), revealed that the IAV in UK land was approximately 6% based on 1997 UK land measurement data, mesoscale modeling and reanalysis data^21^. These findings have been used as the standard IAV of the global wind industry for many years and have played a critical role in the planning and optimization of wind power projects. However, this difference may be overestimated by assuming that the annual mean wind speeds are Gaussian distributed with a standard deviation (σ) of 6% IAV^22^. More recently, based on marine measurements (meteorological mast, LiDAR), reanalysis data, and mesoscale modeling data, DNV was used to calculate IAVs for UK waters in 2016, presuming that Garrad Hassan’s 6% of the IAV may be an overestimation offshore. As a result, DNV revealed that the incidence of IAV in UK waters ranges from 4 to 5.5%^21^. Approximately 4% of the IAV in UK offshore areas has been detected in similar studies^23,24^. Furthermore, the study showed that this improved IAV could lead to a 0.3% reduction in the offshore wind LCOE. However, IAV in UK waters cannot be generalized to other countries or regions because, as indicated above, IAV can depend on topographic conditions, atmospheric stability, and changes in weather patterns.

Lee et al.^25^ stated that monthly and annual variability in wind speed is a key source of uncertainty in the overall wind resource assessment process and emphasized the importance of using rigorous methods to estimate monthly and annual variability. The annual variability in wind resources across the entire continental United States and ocean under current and future climate conditions was assessed, and it was predicted that the variability could reach 10% (increasing or decreasing regionally) based on reanalysis data and regional climate model simulations^11^. Bastin et al.^26^ analyzed the IAV onshore and offshore in India using 30 years of ERA5 reanalysis data and calculated it to be 3.5% and 3.8%, respectively. Pullinger et al.^27^ calculated the IAV over onshore areas in Ireland based on ground observations and reanalysis data and reported that the range of site-specific differences ranged from 4.4 to 6.9%, but an average IAV of 5.4% was a robust estimate and could be used for energy yield assessment. Yu et al.^28^ calculated the long-term IAV of summer wind speed throughout inland China using various types of reanalysis data, and although they showed a considerable difference by region, it was said that the IAV in the reanalysis data tended to slightly underestimate the IAV compared to that in the observation data. In this way, the onshore and offshore emissions of IAV have been evaluated using various types of data for business evaluation and research on onshore and offshore wind farms worldwide. Therefore, the need for advanced measurement and analysis technology to grasp detailed wind speed patterns in various regions is emphasized, and naturally, the same is true for the ROK.

Although the ROK is surrounded by the sea on three sides, the East Sea is characterized by deep water, strong wind speeds, and a relatively uniform coastline. It means the wind speed variation might be relatively lower than West Sea. In contrast, the South Sea and the West Sea, which feature shallow waters, experience relatively lower wind speeds and high turbulence intensity and contain numerous islands, each with distinct wind resource characteristics. Furthermore, typhoons are channeled into the southwestern sea of Korea annually by the Coriolis effect and westerlies, intensifying wind speed variability. These systems typically dissipate in the East Sea after losing latent heat, their primary energy source. Therefore, in this study, the long-term IAV in the Southwest Sea of Korea was calculated, and the characteristics of offshore wind resources were evaluated. The objective is to improve the economic feasibility of projects and decrease LCOE by assessing the potential to reduce uncertainty in future offshore wind projects in the area. This thesis consists of the following steps: First, the status of the Southwest Sea of Korea is introduced, and the meteorological data used were analyzed. Then, an IAV based on reanalysis data is calculated, which presents the results separately for coastal and distant seas and calculates the differences in IAV for different periods of climate data used. Finally, instead of the 6% IAV that has been used as the industry standard, the P90/50 ratio shows how much the uncertainty of offshore wind projects can be quantitatively improved by using the IAV of this study. It is noted that all figures including maps and wind-related spatial distributions presented in the following sections were derived using QGIS v3.38.0 (https://qgis.org/project/overview/).

## Methods

### Analysis area

**Fig. 1:**
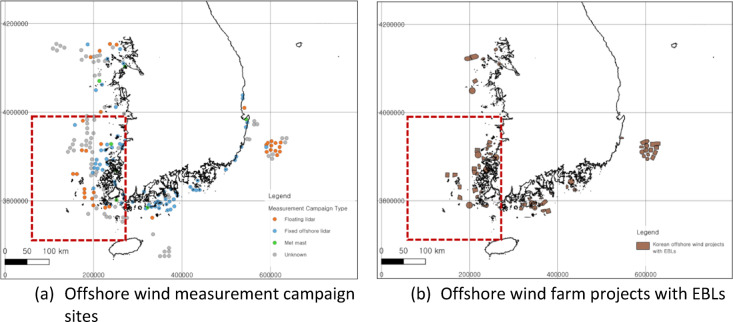
Offshore wind measurement campaign (left, a) and Energy Business License (EBL) status for offshore wind projects (right, b) within the study area of the Southwest Sea of Korea derived from QGIS v3.38.0. (**a**) Floating lidar (Orange), Fixed offshore lidar (Blue), Met mast (Green), Unknown (Gray); (**b**) Offshore wind projects (Brown) that received energy business licenses from Ministry of Trade, Industry and Energy (MOTIE) of South Korea. Coordinate system: EPSG 3857, WGS 84/Pseudo-Mercator.

This analysis focused on the Southwest Sea, specifically in the regions of South Jeolla (Jeonnam) and North Jeolla (Jeonbuk) provinces in the ROK. As of the first half of 2024, more than half of the 90 offshore wind projects that acquired an EBLs were in this region, and this region still has the largest number of offshore measurements in progress or planned in the ROK for offshore wind projects (6). Therefore, the southern area of Gunsan and the northern area of Jindo, the areas where the most attention is concentrated on offshore wind project developers, were selected as target areas of the analysis, as shown in Fig. 1.

### Reanalysis data

**Fig. 2:**
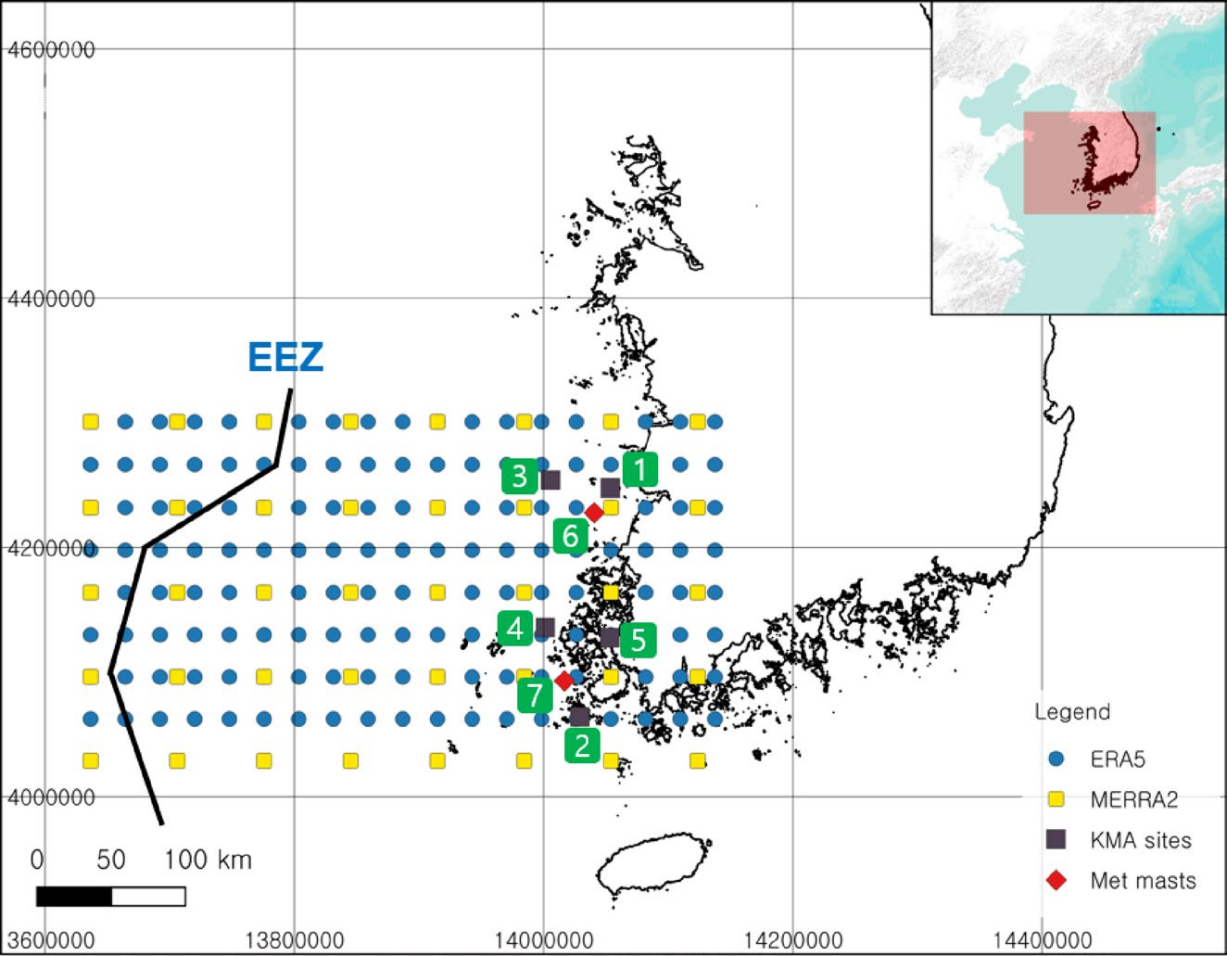
The wind resource data were obtained from the ERA5 + MERRA2 reanalysis dataset and measurement data within the study area derived from QGIS v3.38.0. ERA5 reanalysis data (Blue circles), MERRA2 reanalysis data (Yellow squares), Korea Meteorological Administration’s (KMA) offshore buoys (Black squares) and offshore met masts (Red diamonds) for each node. The Black solid line represents the exclusive economic zone (EEZ) of South Korea. The green numbers represent the location of measurement which were used for validation of IAV result. Coordinate system: EPSG 3857, WGS 84/Pseudo-Mercator.

As part of the analysis, ERA5 and MERRA2 reanalysis data were obtained for each grid point near the exclusive economic zone (EEZ) boundary off the coasts of Jeonnam and Jeonbuk in the ROK. ERA5 and MERRA2 are climate reanalysis data provided by the European Center for Medium-Range Weather Forecasts (ECMWF) and NASA’s Global Modeling and Assimilation Office (GMAO), respectively, and are datasets that reconstruct climate conditions through numerical prediction modeling based on global weather measurement data. MERRA2 has a horizontal resolution of 0.5 degrees × 0.625 degrees (approximately 50 km) by latitude and longitude, and ERA5 has a horizontal resolution of approximately 31 km. In this study, 1-hour average wind speed data for each grid were obtained from a total of 40 years of reanalysis data from 1983 to 2022 (Table 1; Fig. 2).

### Offshore measurement data

To validate the spatial trend of IAV via actual measurements to verify the correlation with reanalysis data, measurement data were collected from offshore buoys and light beacons operated by the Korea Meteorological Administration (KMA) as well as two offshore meteorological masts provided by the Korea Electric Power Research Institute (KEPRI) Research Institute and Mokpo National University (MNU). The locations of the offshore buoys, light beacons, and meteorological records can be found in Fig. 2, and the measurement period, height, and detailed information about the measurement data are shown in Table 1.

**Table 1:** Detailed information on the measurement wind datasets used in this study.

No	Name	Latitude [°]	Longitude [°]	Type	Period	Measurement Parameters	Source
1	Galmaeyeo	35.61	126.24	Light Beacon	2004–2022	Wind speed (WS), wind direction (WD), Temperature (T), relative humidity (RH), pressure (P) (15 m)	KMA
2	Haesuseo	34.26	126.03	Light Beacon	2004–2022	WS, WD, T, RH, P (12 m)	KMA
3	Buan	35.66	125.81	Buoy	2016–2022	WS, WD, T, RH, P (2 m)	KMA
4	Chilbaldo	34.79	125.78	Buoy	2003–2022	WS, WD, T, RH, P (2 m)	KMA
5	Shinan	34.73	126.24	Buoy	2014–2022	WS, WD, T, RH, P (2 m)	KMA
6	Offshore met mast 1	35.47	126.13	Met mast	2014–2016	WS (97, 95, 86, 76, 66, 56, 46, 26 m), WD (95, 76, 56, 46 m), T, RH, P (94, 13 m)	KEPRI
7	Offshore met mast 2	34.48	125.91	Met mast	2016–2018	WS (107, 100, 75, 35 m), WD (107, 96, 35 m), T, RH, P (98, 10 m)	MNU

### Methodology

The sequence of analysis methods used in this study was as follows. First, we Identify the spatial average wind speed distribution and IAV over the past 20 years (2003–2022) and 40 years (1983–2022) for each of the ERA5 and MERRA2 reanalysis datasets. Second, the entire analysis target area was compared by analyzing the IAVs off the coast to determine the difference in the spatial trend characteristics. Third, the quantitative and spatial distribution differences in the IAVs were analyzed over 20 and 40 years for each reanalysis dataset. And then it was analyzed the correlation between offshore measurement data and reanalysis data and analyzed IAV trends based on these data. Finally, we simulated a hypothetical offshore wind farm and performed a sensitivity analysis of the P90/P50 ratio according to each IAV scenario.

First, the average wind speed based on reanalysis data and the spatial distribution of IAVs within the analysis area were identified. This was calculated based on the data for the past 20 and 40 years. Due to the differences in the modeled calculation heights of the available data, the MERRA2 reanalysis data extracted wind speed information at a height of 50 m, and the ERA5 data used wind speed data at a height of 100 m. The IAV was defined as the standard deviation of the annual average wind speed over the entire observation period and was calculated for each node of the reanalysis data.

Second, the spatial distribution characteristics of IAVs were analyzed by defining the sea area as up to 100 km off the coastline offshore for comparison with the entire analysis target area up to the EEZ. This is because most bottom-fixed offshore wind projects in the Southwest Sea of Korea are located within 100 km of the coastline, within a water depth of 60 m. Therefore, it was necessary to determine what differences the characteristics of offshore IAVs exhibit from the perspective of bottom-fixed offshore wind projects.

Third, this study attempted to quantitatively and spatially determine the distribution characteristics of the IAVs over the past 20 and 40 years for each reanalysis dataset. The aim of this analysis was to determine the potential indirect impacts of recent climate change on the variability in annual wind speeds in the Southwest Sea of Korea.

Although the characteristics of IAVs in the Southwest Sea of Korea based on reanalysis data can be identified through steps 1 to 3, their validity is assessed through linear regression correlation analysis with the actual measurement data, given that the selected measured datasets were not assimilated into the numerical prediction model of the reanalysis datasets. Therefore, the correlation of the reanalysis data against the measurement data was analyzed using the measurement data of two offshore meteorological masts provided by KEPRI and MNU along with the measurement data of offshore buoys and light beacons operated by the KMA. Particularly in the case of offshore buoys and light beacons, the measurement period was as long as 10 years at certain locations. Therefore, this study aimed to analyze the characteristics of IAV distribution using measurement data to determine whether the IAV trends are consistent with those observed in the reanalysis datasets.

Different heights were utilized for verification and calculation due to the availability and limitations of each dataset. While quantitative IAV values were expected to vary with height, it was determined that spatial trends remain consistent regardless of altitude. Accordingly, this informed our methodological decision to emphasize spatial coherence in our analysis.

Finally, an annual energy production (AEP) assessment is conducted by creating a hypothetical offshore wind farm within the analysis area. Based on several IAV scenarios that include IAV values calculated in the Southwest Sea of Korea, simulations on how the P90/P50 ratio might vary were performed. The P90/50 ratio in AEP is an important indicator of uncertainty in predicting the energy production of wind farms. In this context, P50 and P90 are defined as follows:

- P50: The probability of exceedance of 50% is the median expected annual AEP, meaning that there is a 50% probability that the annual energy production will be higher than this value for any year across the operational lifetime of the wind farm.
- P90: The probability of exceedance of 90% is a conservative estimate, meaning that there is a 90% probability that annual energy production will be higher than this value for any year across the operational lifetime of the wind farm.

The P90/P50 ratio is an essential tool for evaluating the predictive reliability of wind power projects. This allows investors and financial institutions to better understand the risks and potential returns of the project, with a ratio closer to 1 indicating that the uncertainty associated with the energy yield prediction is lower. The ultimate objective of this study is to demonstrate how long-term IAVs in the Southwest Sea of Korea, calculated from reanalysis data and validated by measurement data, can reduce the uncertainty in energy yield assessments for offshore wind projects, offering a more accurate assessment than generic assumptions such as the historical 6% IAV.

## Results

### Change trend of the wind speed

**Fig. 3:**
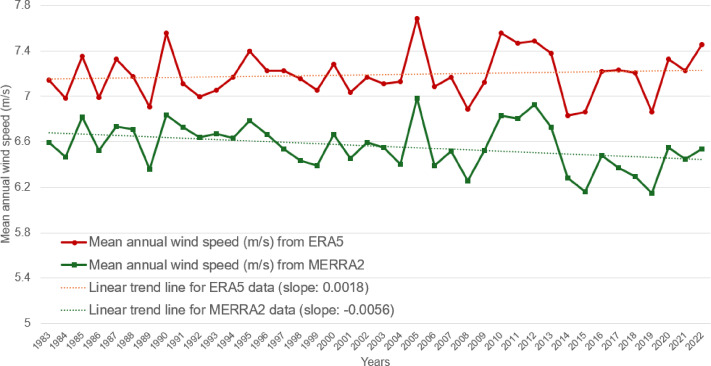
Wind speed fluctuations based on the reanalysis datasets (ERA5 and MERRA2) on the central node of the study area. The ERA5 long-term wind speed at 100 m height (Red line) represents the slightly increasing slope (0.0018) whilst the MERRA2 long-term wind speed at 50 m height (Green line) represents the slightly decreasing slope (−0.0056) during the period of 40 years.

**Fig. 4:**
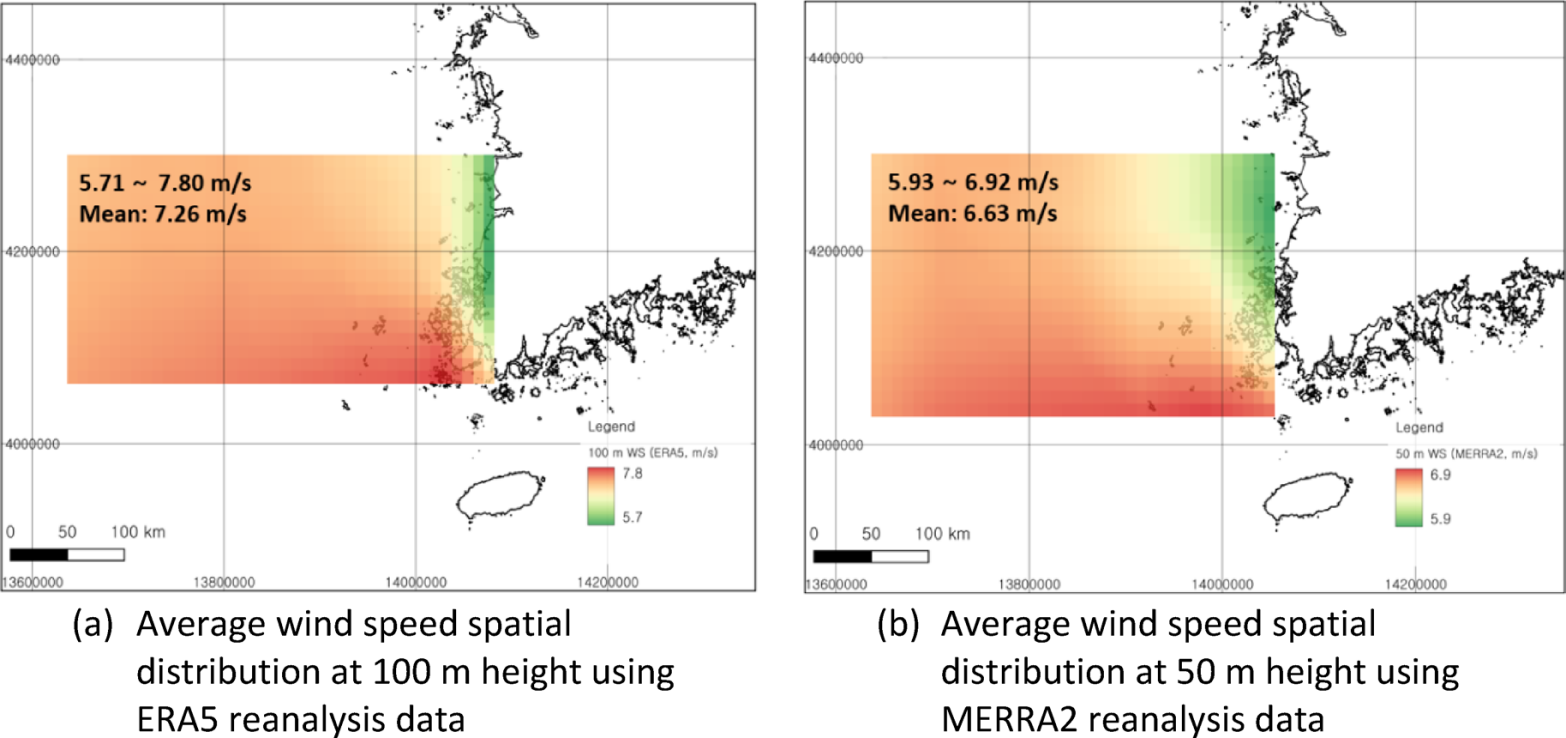
Map of the spatial distribution of 20-year long-term average wind speed using reanalysis data within the study area derived from QGIS v3.38.0. (**a**): The spatial horizontal distribution of 100 m height long-term wind speed based on ERA5 reanalysis data. (**b**): The spatial horizontal distribution of 50 m height long-term wind resources based on MERRA2 reanalysis data. The coordinate system used was an EPSG 3857 and a WGS84/Pseudo-Mercator.

Figure 3 shows the change trends in the ERA5 and MERRA2 wind speeds based on long-term reanalysis data from random nodes located in the middle of the analyzed offshore area (ERA5: 35.250°N, 125.500°E; MERRA2: 35.000°N, 125.625°E). Examining the ERA5 long-term trend of wind speed changes in the Southwest Sea of Korea over the past 40 years reveals a slightly positive average slope, suggesting a marginal increase in average wind speed over time. Conversely, the MERRA2 reanalysis data indicate a weak negative slope, implying that the wind speed at a height of 50 m has decreased compared to that in the past. Figure 4 shows the mean wind speeds across the more recent 20 years of data. This figure indicates the spatial variation in the mean wind speeds across the observed region.

### IAV spatial distribution

**Fig. 5:**
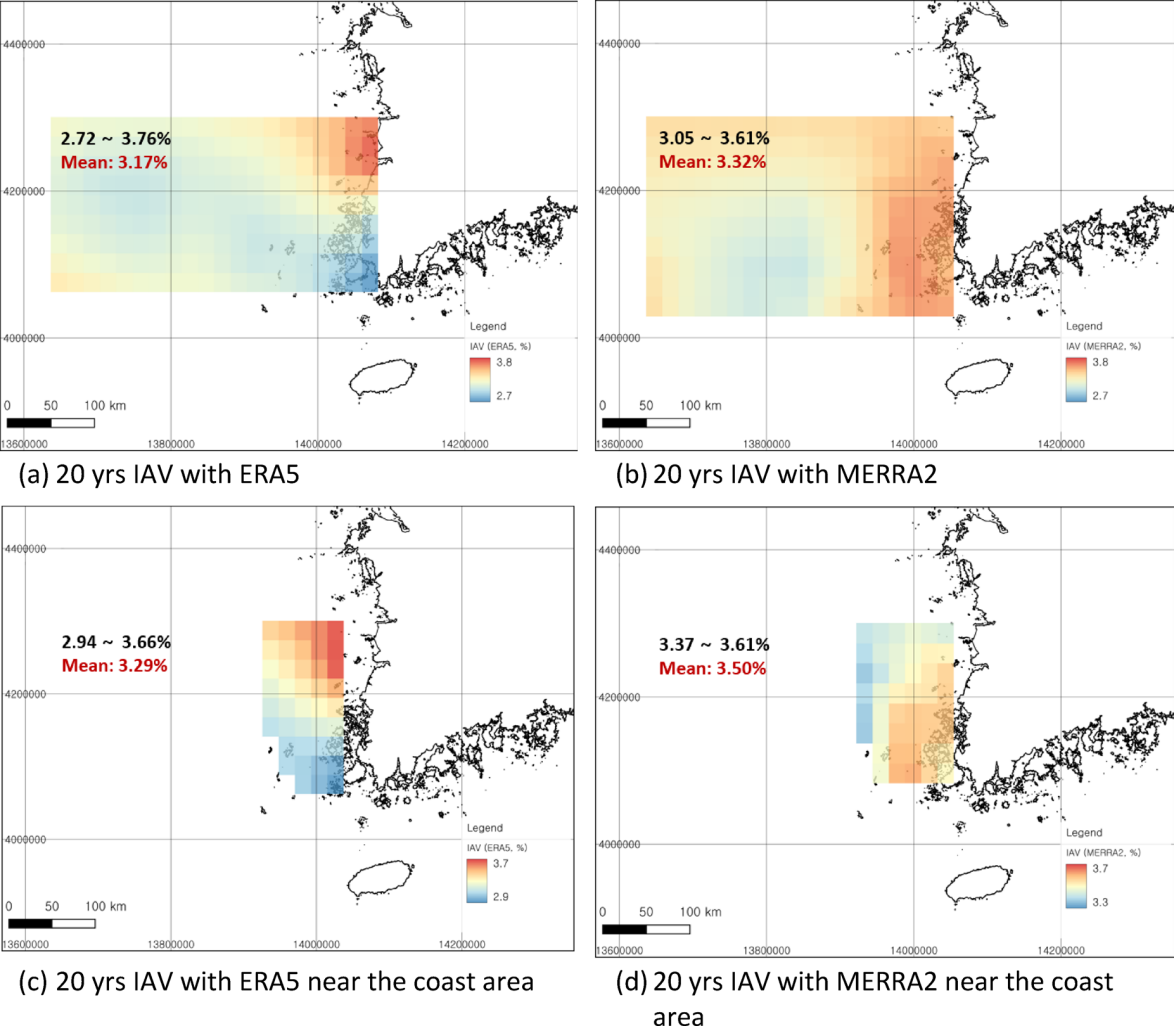
Spatial distribution of IAV using reanalysis data (ERA5 and MERRA2) within the study area derived from QGIS v3.38.0. (**a**) IAV with ERA5 reanalysis data for 20 yrs; (**b**) IAV with MERRA reanalysis data for 20 yrs; (**c**) IAV with ERA5 reanalysis data for 20 yrs within 100 km away from the coast; (**d**) IAV with MERRA2 reanalysis data for 20 yrs within 100 km away from the coast. Coordinate system: EPSG 3857, WGS 84/Pseudo-Mercator.

Based on long-term climate data from the past 20 and 40 years of ERA5 and MERRA2 reanalysis data, the spatial distribution of IAVs in the Southwest Sea of Korea was calculated (Fig. 5). Both data sources showed relatively greater IAVs in coastal areas than in the distant sea. However, for the ERA5 data, high IAVs were observed along the Jeonbuk coast in the northeastern region of the analysis area, while lower IAVs were observed along the southeastern coast of Jeonnam, specifically in Sinan-gun. As shown in Fig. 4, this may be because the wind speed in ERA5 is greater in Jeonnam than in Jeonbuk, and in general, the turbulence intensity is greater when the wind speed is lower. Considering that both the turbulence intensity and the IAV yield a standard deviation from the mean, the variability is greater in Jeonbuk, where the wind speed is lower.

In comparison, MERRA2 also indicates relatively low wind speeds in Jeonbuk as shown in Fig. 4, but does not exhibit a pronounced spatial difference in IAV (Fig. 5b). This lack of distinction in MERRA2 appears to result from the generally lower wind speeds at 50 m height, the presence of numerous islands in the southeastern region (Jeonnam) that contribute to complex terrain, and the relatively lower data height in that area than ERA5 of 100 m height. Accordingly, IAV derived from MERRA2 tends to be significantly higher in the southeastern region (Jeonnam), where islands are densely concentrated and terrain complexity is substantial.

Since most of the bottom-fixed offshore wind projects in the analyzed area are either underway or planned within 100 km of the coastline, the IAV in this offshore region was closely examined. The IAV near the coast was slightly greater than the IAV for the entire analysis area (Table 2).

**Table 2:** IAV differences between analysis periods in near-coastal and offshore areas.

IAV	ERA5 [%]	MERRA2 [%]	ERA5 (near offshore) [%]	MERRA2 (near offshore) [%]
IAV (20 yrs) (2003–2022)	3.17	3.32	3.29	3.50
IAV (40 yrs) (1983–2022)	2.78	2.79	2.82	2.94

### Differences in IAV spatial distribution according to analysis period

**Fig. 6:**
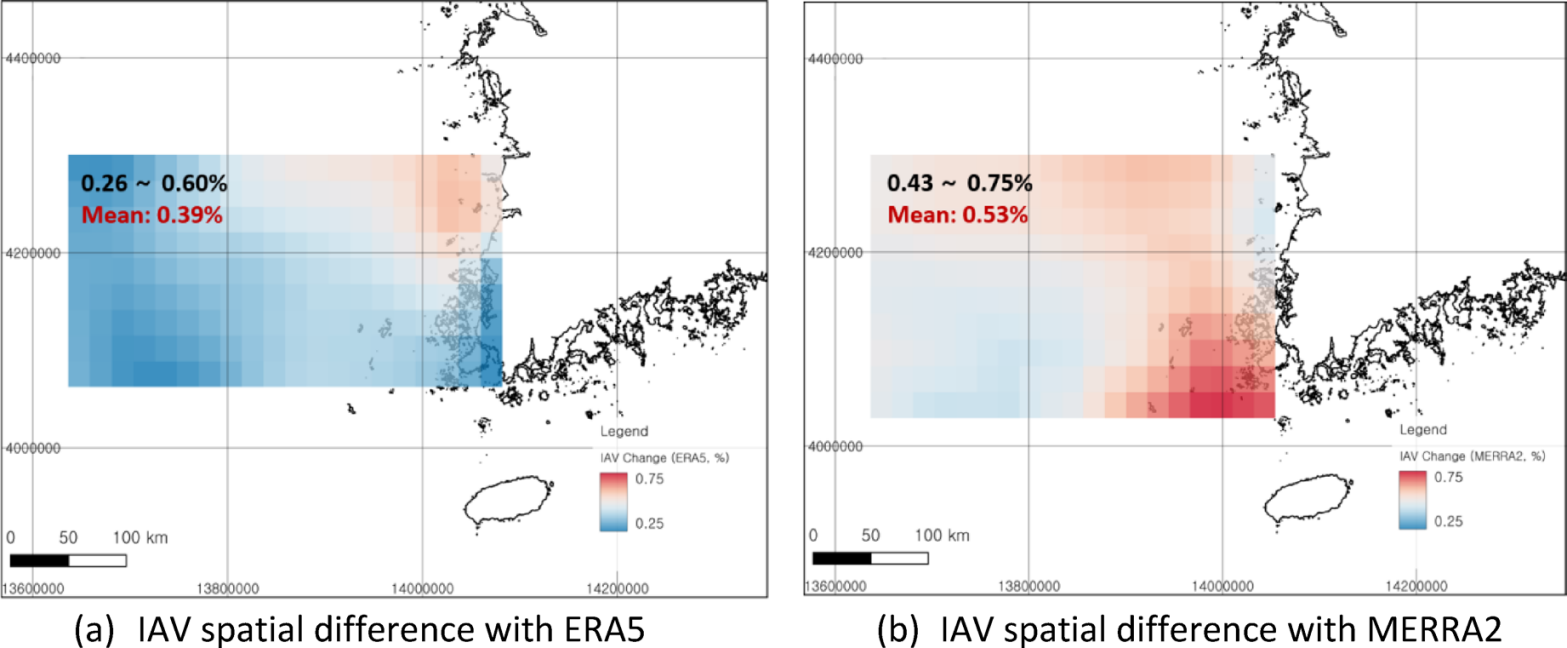
Differences in the spatial distributions of IAVs between the 20-year and 40-year long-term datasets derived from QGIS v3.38.0. (**a**) Difference between 20 yrs IAV and 40 yrs IAV using ERA5 reanalysis data (mean: 0.39%); (**b**) Difference between 20 yrs IAV and 40 yrs IAV using MERRA2 reanalysis data (mean: 0.53%). Coordinate system: EPSG 3857, WGS 84/Pseudo-Mercator.

The IAVs for the last 20 years were calculated to be much greater than those for the last 40 years (Fig. 6). These characteristics were consistent for both the MERRA2 and ERA5 datasets, suggesting that the meteorological or marine environment may have undergone long-term changes due to climate change. On the other hand, the results of the MERRA2-based analysis suggest that the IAVs in Sinan-gun, southeast of the analysis area, have increased significantly over the past 20 years. Unlike ERA5, MERRA2 utilizes wind speed data at a height of 50 m, and it is necessary to consider that it is a complex wind climate region with a considerable number of islands distributed in comparison to the northeast.

### Verifications with offshore measurement data

Since the reanalysis data are produced by a numerical prediction model, variations from the actual measurement data occur. Therefore, to understand the reliability of the IAV calculated from reanalysis data, it is essential to verify the correlation between the reanalysis data and the measurement data and observe similarities in patterns.

**Fig. 7:**
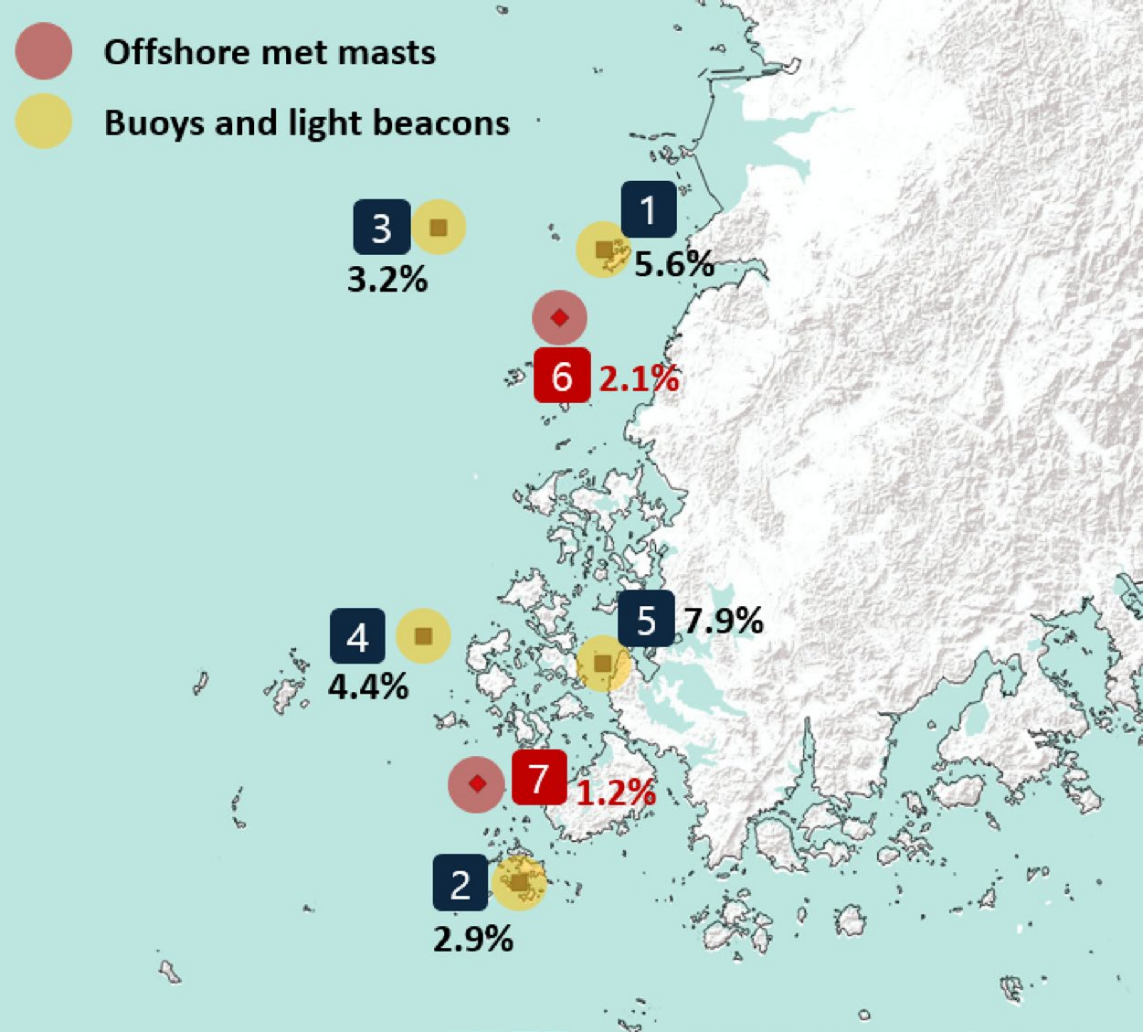
IAVs for each offshore measurement site within the study area derived from QGIS v3.38.0; yellow shaded circles are KMA measurement sites (buoys and light beacons) with 2–15 m height. The red shaded circles represent offshore met masts with a height of approximately 100 m. (Bouy) 1-Galmaeyeo: 5.6%; 2-Haesuseo: 2.9%; 3-Buan: 3.2%; 4-Chilbaldo: 4.4%; 5-Shinan: 7.9%; (Met mast) 6-mast 1: 2.1%; 7-mast 2: 1.2%.

Measurement data from years with a less than 90% data recovery rate were excluded from the analysis, and the IAVs for each measurement site are shown in Fig. 7. The wind speed correlation between the offshore measurement data and the reanalysis data showed that the correlation (coefficient of determination, R²) of ERA5 against the measurement data ranged from 0.60 to 0.85, with the highest and very good correlation occurring at offshore met mast 1 (No. 6) and the lowest correlation occurring at Chilbaldo (No. 4). The spatial pattern of IAV was quantitatively similar to ERA5-derived patterns. This agreement remains significant specifically for IAV, even when considering the height discrepancy between offshore measurements (∼10 m) and ERA5 model data (100 m, Table 3). However, this height consistency applies primarily to IAV analysis. For wind speed correlations (R²), ERA5 shows substantially stronger alignment with mast measurements than with buoy data. Meanwhile, MERRA2 correlations with measurements ranged from 0.57 to 0.78—notably lower than ERA5. Consequently, while ERA5 demonstrates higher reliability for IAV patterns, its wind speed accuracy remains height-dependent.

On the other hand, in the case of offshore meteorological masts, the measurement period of the two pieces of equipment was less than three years, so the resulting IAV figures are not considered representative. Nevertheless, the offshore met mast datasets have proven useful for validation because it was possible to confirm that the correlation with ERA5 was greater than 0.8 after checking the sensor measurement data at heights of approximately 100 m and 50 m.

**Table 3:** The correlation and IAV data are based on reanalysis and offshore measurement datasets.

No	Name	Measurement height [m]	*R*² with reanalysis data		IAV			Available period
			ERA5 [-]	MERRA2 [-]	Meas. [%]	ERA5 [%]	MERRA2 [%]	
1	Galmaeyeo	15	0.714	0.690	5.58	3.88	3.54	13.4 yrs
2	Haesuseo	12	0.676	0.667	2.92	3.07	3.59	16.2 yrs
3	Buan	2	0.713	0.695	3.16	2.65	1.97	6.9 yrs
4	Chilbaldo	2	0.606	0.619	4.41	2.71	2.95	13.6 yrs
5	Shinan	2	0.625	0.574	7.90	2.43	2.09	8.8 yrs
6	Offshore met mast 1	95	0.829	0.773*	2.15	3.73	3.56	2.8 yrs
7	Offshore met mast 2	100	0.811	0.753*	1.18	2.99	3.55	2.7 yrs

*R^2^ with MERRA2 reanalysis data was calculated with 56 m height for mast 1, 35 m height for mast 2.

### P90/P50 ratio based on energy yield

A hypothetical offshore wind farm with a capacity of 510 MW was simulated within the analysis area to evaluate the annual energy production. The NREL 15 MW 240 RD WTG model was used, with a hub height of 150 m and 34 WTGs. The WTGs were spaced at 8 rotor diameters (RDs) (1920 m) and 6 RDs (1440 m) in the prevailing wind direction and the cross-wind direction, respectively. The Vortex MAPS (150 m height) mesoscale modelling data which has 1 km spatial resolution with 20-year period was used for energy yield assessment. The wake losses were estimated with the Jensen wake model^29,30^for which the wake decay constant was 0.04^31,32^. Secondary losses were applied to derive the P50 value (considering wake effects, non-availability, electrical, environmental, WTG performance, curtailment and other: total 15.0%). The uncertainties were then applied based on generic industry standards. However, the uncertainty associated with long-term climate representation was assessed using four IAV scenarios for sensitivity analysis (Table 4, Eq. 2).

2 $$\begin{aligned} Historical{\text{ }}period{\text{ }}uncertainty = \frac{{IAV}}{\sqrt{N}}{\text{ }}[\% ]\\N:{\text{ }}hindcast{\text{ }}years\\ \end{aligned}$$

When calculating AEP (Annual Energy Production), the P90/P50 ratio is an important indicator of project uncertainty and risk. It can be generally described as follows (units are dimensionless):

- When the ratio is 0.9 or higher, the project uncertainty is considered low and stable.
- A ratio between 0.8 and 0.9 indicates a moderate level of uncertainty.
- A ratio below 0.8 indicates high uncertainty and may require additional analysis and caution.

Assuming that the traditional 6% IAV is the base case, the P90/P50 ratio improved by 0.1%, 0.3%, and 0.4% when IAVs of 5.5%, 4%, and 3%, respectively, were applied. Given that the average IAV concentration in the Southwest Sea of Korea, as estimated in this study, is approximately 3%, there is potential for a substantial reduction in uncertainty, which warrants further investigation.

**Table 4:** Summary of the IAV sensitivity analysis results for calculating the P90/P50 ratio.

No	Scenario	Uncertainty of long-term representation [%]			Change in P90/P50 compared to IAV case A	
		1 yr	10 yrs	20 yrs	%	P90/P50
A	IAV 6%	6.0	1.9	1.3	–	0.903
B	IAV 5.5%	5.5	1.7	1.2	+ 0.10	0.904
C	IAV 4.0%	4.0	1.3	0.9	+ 0.28	0.905
D	IAV 3.0%	3.0	0.9	0.7	+ 0.41	0.907

## Conclusions

The IAV in the Southwest Sea of Korea was analyzed using reanalysis data, which was verified with offshore measurement data. These results confirmed how much the uncertainty of offshore wind projects in the region could be improved. The IAVs based on the MERRA2 and ERA5 reanalysis data were 3.32% and 3.17%, respectively, in the Southwest Sea of Korea over the past 20 years, while the IAVs in the 40-year period were 2.79% and 2.78%, respectively. Relatively greater IAVs were observed in the coastal areas, with 3.50% (MERRA2) and 3.29% (ERA5) for 20-year IAVs and 2.94% (MERRA2) and 2.82% (ERA5) for 40-year IAVs. Although the height of each reanalysis dataset must be considered, the MERRA2-based IAV yields higher values ​​overall near the coast than does the ERA5-based IAV. The correlation analysis between the reanalysis data and offshore measurement data indicates that ERA5 performs well, showing a greater correlation with the measurements than does MERRA2. Although the general spatial trend of the IAV based on offshore measurement datasets is similar to that of ERA5, measurements from Chilbaldo and Shinan suggest that ERA5 may not fully capture the complexity of coastal regions due to its coarse resolution. A site-specific IAV assumption in southwestern Korea might be more applicable than the historical 6% IAV assumption. With respect to the hypothetical offshore wind farm, the P90/P50 ratio improved by 0.4% as a result of applying an IAV of 3% in this study compared to the traditional 6% IAV or 4-5.5% in the UK offshore region.

Reducing the uncertainty of wind power has positive economic, technological, and environmental benefits and can play a vital role in increasing the success and sustainability of wind power projects. This study provides an important basis for confirming whether the trend in wind speed variations based on reanalysis data can be used for actual uncertainty analysis when long-term measurement data cannot be obtained at sea. However, it is noted that this IAV result based on historical data in IAV is likely to accumulate climate change-induced biases over the 25–30 years of typical offshore wind farm operation period (life-cycle). So it is worth conducting further analysis based on RCP or SSP scenarios, it could take into account the non-stationary changes in wind speed patterns due to climate change.

In addition, this study can contribute to improving the quality of basic research related to the ROK’s offshore wind resources and climatological characteristics to reduce the uncertainty of offshore wind projects and project risks.

## Data Availability

The MERRA-2 reanalysis dataset is available at MDISC, managed by the NASA Goddard Earth Sciences (GES) Data and Information Services Center (DISC) and can be downloaded through https://disc.gsfc.nasa.gov/datasets? project=MERRA-2. The ERA5 reanalysis dataset is available at the Copernicus Climate Data Store at https://cds.climate.copernicus.eu/cdsapp#!/dataset/reanalysis-era5-single-levels? tab=overview. The offshore measurement data (buoys, light beacons) are publicly available by the Korea Meteorological Administration (KMA) and can be downloaded at https://data.kma.go.kr/data/sea/selectBuoyRltmList.do? pgmNo=52. The offshore meteorological mast measurement data is not publicly available since those are the property of the Mokpo National University and Korea Electric Power Research Institute but are permitted to be used for this paper by Professor Chae-Joo Moon, and KEPRI.
